# Impact of the COVID-19 Pandemic on Medical Oncology Workload: A Provincial Review

**DOI:** 10.3390/curroncol30030238

**Published:** 2023-03-07

**Authors:** Margaret Sheridan, Bruce Colwell, Nathan W. D. Lamond, Robyn Macfarlane, Daniel Rayson, Stephanie Snow, Lori A. Wood, Ravi Ramjeesingh

**Affiliations:** Division of Medical Oncology, Department of Medicine, Nova Scotia Health and Dalhousie University, Halifax, NS B3H 2Y9, Canada

**Keywords:** medical oncology, COVID-19, workload, virtual care

## Abstract

(1) Background: Cancer is the leading cause of death in Canada, with significant resource limitation impacting the delivery of cancer care nationwide. The onset of the COVID-19 pandemic forced additional resource restriction and diversion, further impacting care delivery. Our intention is to analyze the impact COVID-19 on a provincial medical oncology workload and bring attention to the limitations of the current workload metric for oncologists. (2) Methods: All medical oncology patient encounters were extracted and compared, collected by year and encounter type, from April 2014 through March 2022. (3) Results: There was an increase in all patient encounters by an average of 9.5% per year, including during the strictest COVID-19 restrictions. There was an increase in virtual care encounters from 37.9% to 52.1%. (4) Conclusions: Medical Oncology workloads have increased over time and estimates suggest growing demand. Little data exist to inform workforce requirements and actual workload is not captured by the current metric. Though volume of new consults continues to increase, COVID-19 has highlighted additional changes in the delivery of care, likely with lasting impact, little of which are included in the current workload metric.

## 1. Introduction

Cancer is the leading cause of death in Canada, with increasing incidence and mortality [1]. Almost half of Canadians will be diagnosed with cancer in their lifetime and incidence is projected to increase a further 79% by 2032 [1]. Eastern and Central Canada are found to have the highest predicted incidence rates, with the second highest age-standardized provincial rates observed in the province of Nova Scotia [2]. Nationally, Cancer Care programs face significant resource limitations, including personnel, infrastructure, and escalating treatment expenditures, all of which were exacerbated due to the COVID-19 pandemic [3]. Workforce evaluation and planning, as well as adaptability, is critically important to the provision of optimal patient care and current metrics do not reflect the full scope of medical oncology practice, nor the lasting changes from the COVID-19 pandemic.

Currently, there are very limited published data on Medical Oncology workload benchmarks and even less on actual workload. The most often cited metric in the Canadian literature is defined as number of new cancer patient consultations per oncologist per year [4]. A recent global study, which included a Canadian cohort, was conducted to address medical oncology workload and describe allocation of resources [5]. Two other reports published by Canadian groups have attempted to identify flaws in the current medical oncology workforce assessment model, suggesting a change is needed [4]. The authors emphasized the importance of developing a comprehensive workload metric to account for escalating complexity of care and evolving treatment landscapes more accurately [6]. These studies represent the only data available in the Canadian context since the Cancer Care Ontario Systemic Therapy Task Force report was published in 2000 [7]. 

While the current demands are already straining the system—secondary to Canada’s aging population—including longer cancer patient survival on systemic treatment [8] and availability of novel treatments, the COVID-19 pandemic has exacerbated this further. COVID-19 has significantly impacted all healthcare systems and delivery of care, with federal and provincial governments recommending variable safety measures, including limitations on in-person encounters when possible, and a shift toward provision of virtual care. Both surgical and radiation oncology have faced significant limitations in care delivery at times throughout the pandemic, including cancelations and operating room closures due to over-capacity hospital admissions, physician discretion, staff shortages due to COVID-19, and cancelation of regular radiation consultation and treatment, in an effort to minimize exposure and maximize patient safety [3]. Across Canada, there was a 20% reduction in cancer surgeries in the first 6 months of the pandemic, as per data collected by the Canadian Institute for Health Information [9]. This increased the demand on medical oncology, for example, with the need for introduction or continuation of chemotherapy, as opposed to surgical resection in some cases [10,11,12]. Though numeric data reflecting that specific impact has been difficult to collect, this has been reported by most Medical Oncologists practicing in Canada and has been noted globally [9,13].

With the lack of published information on medical oncology workload, we devised a study to review what the current workload for medical oncologists is in the Nova Scotia Central Zone, which accounts for the greatest portion of the province’s cancer care delivery. We ask whether the workload has evolved over time and what the impact of the COVID-19 pandemic was on the workload, if any. We hope this may inform a comprehensive framework to capture workload that could aid in future discussions on resource allocation and workforce planning in Canada. 

## 2. Methods

The Nova Scotia Cancer Centre within the Nova Scotia Health Central Zone (NSHCZ), affiliated with Dalhousie University, is the academic center for the province of Nova Scotia, Canada, providing consultative and ongoing cancer care for approximately 72% of the cancer patients in the province, a total population of just over 1,000,000 people. It accounts for 80% of the provincial medical oncologists (MO), while the remaining 20% practice in the 2 other geographically separated centers in the province. All are under the jurisdiction of the Cancer Care Nova Scotia Program (CCNSP) and have a similar clinical practice.

Data were extracted for all patient encounters from the Oncology Patient Information System (OPIS). OPIS is the electronic booking system for all patient encounters at the Nova Scotia Cancer Center. Encounter types included are new patient consultations, follow-up visits, which could be undertaken in person or virtually, and outpatient treatment visits. Additionally, we captured telephone toxicity assessments (nursing-led phone calls to patients of approximately 10 min duration to review treatment toxicities, then reviewed and approved by the Medical Oncologist to authorize subsequent treatment cycles) and chart reviews (scheduled opportunities for the physician to review, discuss, and follow-up on patient investigations or clinical status; these often include patient phone calls, but are not intended as full follow-up appointments). Both in-person and virtual encounters are captured through OPIS and data were included for the full complement of 12–16 full time Medical Oncologists (the number of staff increased over the study period) and one physician extender (in this case a nurse practitioner with extra training in oncology, who provides clinical support), as well as the resident trainees in oncology, in the outpatient setting over the study period. 

Encounter data were collected per the Nova Scotia Health fiscal year. The fiscal year begins 1 April and ends 31 March of the following calendar year. We included data from 1 April 2014 to 31 March 2022 (thus recorded as fiscal years 2014–2021), including the duration of the first provincially mandated pandemic-associated restrictions in March 2020. The total number of encounters was extracted and sorted by encounter type for each year. As the OPIS system does not categorize patient encounters by tumor type, to assess for this, each encounter was categorized into disease site based on the tumor sites treated by the individual physician. Thus, a physician who treats 50% breast and 50% GI had their patient encounters split in half, as an approximation of the workload for each disease site. 

## 3. Results

### 3.1. Overall Clinical Workload

**Figure 1:**
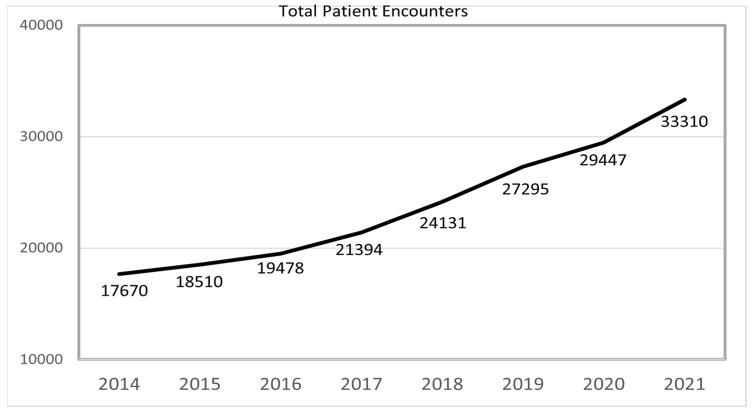
Total patient encounters from 2014 to 2021.

The total number of patient encounters, including new consults, follow up visits, telephone toxicities, and chart reviews were analyzed from 2014 to 2021. Overall, there were 17,670 patient encounters in 2014, compared to 33,310 in 2021, translating to an average annual increase in total encounters of 9.5%. (Figure 1) In 2014, there were 2228 new patient consults, increasing to 3254 by 2021 (a 46% increase). The number of follow-up visits increased from 8902 to 13,485 (52% increase). Chart reviews and telephone toxicities increased by 146% and 200%, respectively, (5625 chart reviews in 2014 compared to 13,822 in 2021 and 915 telephone toxicities in 2014 compared to 2749 in 2021. (Table 1) To consider the change in the number of medical oncologists located in the NSHCZ over time, we analyzed the number of patient encounters per medical oncologist (PEMO). In 2014, there were 12 medical oncologists (MO) and 1 nurse practitioner (NP) in the NSHCZ, leading to a 1359.2 PEMO. By 2021, there were 15 MOs and 1 NP in the NSHCZ, which led to a 53.2% increase in the PEMO (2081.9). This increase is primarily the result of the increase in chart checks per MO (432.7 in 2014 and 863.9 in 2021; 99.6% increase) and telephone toxicities per MO (70.4 in 2014 and 171.8 in 2021; 144% increase). The number of new consults per MO increased by 18.7% (171.4 in 2014 and 203.4 in 2021), with an increase in follow-up visits per MO of 23.1% (684.8 in 2014 and 842.8 in 2021). 

**Figure 2:**
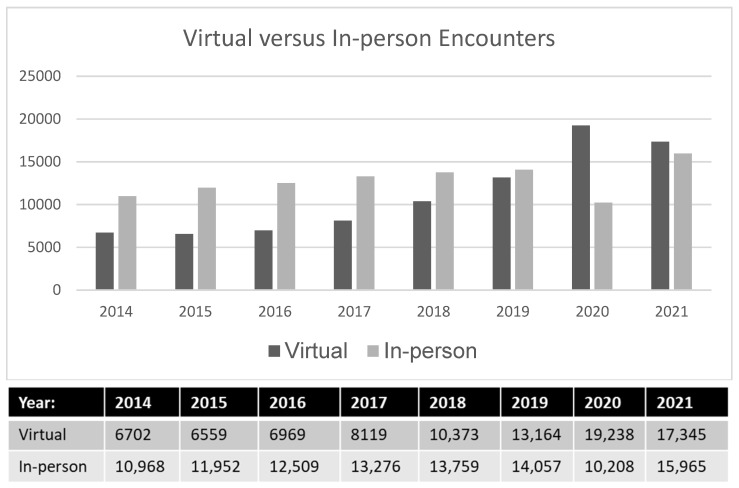
Total encounters from 2014 to 2020, separated by virtual and in-person encounters.

Figure 2 presents the annualized data for both in-person and virtual care encounters, demonstrating increases across all encounter types. In-person encounters increased by 45.6%, from 10,968 to 15,965, between 2014 and 2021. Within the same timeframe, virtual care encounters, including virtual follow-up appointments, telephone toxicities, and chart reviews, grew more substantially, from 6702 to 17,345 (158.8% increase). Of note, even before the pandemic, virtual encounters were rising annually (2017–2018 = 16.5% increase, 2018–2019 = 27.7% increase, 2019–2020 = 26.9% increase) when compared to in-person encounters (2017–2018 = 6.1% increase, 2018–2019 = 3.6% increase, 2019–2020 = 2.2% increase). This clearly suggests that virtual care provision was increasing even prior to the COVID-19 pandemic. 

### 3.2. Clinical Workload during the COVID-19 Pandemic

There was a 7.9% increase in overall patient encounters between 2019 and 2020 (29,447 in 2020 compared to 27,295 in 2019). Although virtual encounters appeared to be on the rise prior to the pandemic, there was a 46% increase in virtual care encounters from 2019–2020, with a corresponding decrease in in-person encounters over this period (27%). As the height of the first and second waves of the pandemic settled in Nova Scotia, we saw a return of in-person encounters (15,965 in 2021, 10,208 in 2020, and 14,057 in 2019), with a small reduction in virtual care when compared to 2020 (17,345 in 2021 and 19,238 in 2020). There was still a 31.8% increase in virtual care when compared to 2019 (17,345 in 2021 to 13,164 in 2019).

Overall, the greatest number of patient encounters in 2020 were chart reviews, followed by follow-up visits, new consultations, and telephone toxicities. All types of encounter had increased, with the greatest percentage increase noted in chart reviews (virtual) and follow-ups (some in-person, some virtual) across the study timespan, regardless of tumor/disease site. This was maintained in 2021.

### 3.3. Clinical Workload by Tumor Site

**Figure 3:**
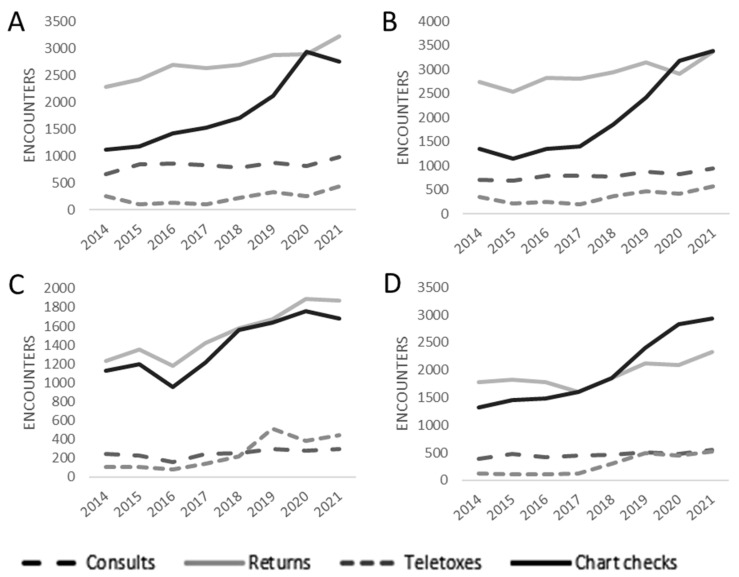
Workload by Disease Site. Consults, follow-ups, telephone toxicities, and chart check encounters from 2014–2020 for (**A**) Gastrointestinal cancers, (**B**) Breast cancers, (**C**), Genitourinary cancers, and (**D**) Lung cancers.

To look at the changes in workload over time by disease site, we separated each patient encounter by attending physician and subsequently categorized them into four major tumor sites (breast, gastrointestinal (GI), genitourinary (GU), and lung). Note, encounters for skin, central nervous system, and sarcoma were excluded from this analysis. Additionally, it should be emphasized that these are estimates based on the treating physician’s typical patient load. When you look at total volume, no matter which disease site is looked at, there is an increase in all four of the workload metrics. GI cancer treaters saw an increase from 2014 to 2021 in total consults (669.2 to 975.5), return visits (2284 to 3227.4), telephone toxicities (257.2 to 440), and chart checks (1121.3 to 2749.2) (Figure 3A). Breast cancer treaters saw an increase from 2014 to 2021 in total consults (712.3 to 949), return visits (2740.8 to 3370.8), telephone toxicities (343.5 to 577), and chart checks (1343.5 to 3382) (Figure 3B). GU cancer treaters saw an increase over the same time frame in total consults (247.75 to 298), return visits (1228.25 to 1876.8), telephone toxicities (104 to 443), and chart checks (1130 to 1682) (Figure 3C). Lung cancer treaters saw an increase in total consults (392.8 to 558.3), return visits (1773.8 to 2324.7), telephone toxicities (343.5 to 525.8), and chart checks (1343.5 to 2931.1). 

As the number of MOs changed from 2014 to 2021, we determined the number of each encounter type seen per MO by each disease site. In both breast and GU disease sites, we saw a decrease in 2021 in the consults seen per MO and a decrease in follow-up visits in breast cancer seen, likely reflective of the COVID-19 pandemic (Table 2). There were increases in consult and follow-up visits in both GI (consults 9.3%, follow-up visits 6.0%) and lung disease sites (consults 13.7%, follow-up visits 4.8%). Moreover, three disease sites saw an increase in chart checks (GI: 83.9%; Breast:76.2%; Lung: 72.6%), while GU saw a slight dip in chart checks per MO (0.8%). All four disease sites saw an increase in telephone toxicities per MO (GI: 28.4%; Breast:17.5%; GU: 184.0%; Lung: 224.6%.). To estimate the work one consult generates, we compared the number of follow-up visits, telephone toxicities, and chart reviews to the number of consults in 2014 and 2021. A GI consult led to a 1.9 chart review increase in 2021, when compared to a GI consult in 2014, but no change in follow-up visits or telephone toxicities. A breast consult saw a similar 1.7 chart review increase in 2021, with no change in the number of follow-up visits or telephone toxicities. A lung consult led to a 1.9 increase in chart reviews and a 0.6 increase in telephone toxicities per consult in 2021, but no change in follow-ups. GU on the other hand, saw an increase of 1.3 visits, 1.1 telephone toxicities, and 1.0 chart reviews per consult. 

## 4. Discussion

This study has attempted to provide an account of the changes in MO workload over time and to review the impact of the COVID-19 pandemic on the workload in hopes of aiding in future discussions on resource allocation planning in Canada. The Systemic Therapies Task Force was established in 2000 by Cancer Care Ontario (CCO) and determined the workload benchmark for Canadian Medical Oncologists based on an optimal average annual target of new patient consults per practitioner. This target was originally derived by calculating the number of hours of patient care available per academic Oncologist per year divided by the number of hours estimated for patient care required per specific tumor type [7]. This has been the metric used nationally since then to measure workload and inform cancer program hiring practices. According to the study by Fundytus et al., high-income countries, including Canada, seemed to fall within the target range for new consults. However, it is important to note that although the median reported clinical volume was consistent with the proposed annual target, half of the survey participants exceeded it [4]. A recent snapshot assessment of medical oncology workload in Europe showed Western European countries would see on average 175 consults per year, similar to the Canadian standard, while Eastern European countries saw an average of 225 consults in a year [14]. We believe we are the first to look at the different encounter types for medical oncologists in Canada. 

Our data suggest an increase in volume of patient encounters over time. No matter which metric was reviewed, the total volume of consults, follow-up visits, telephone toxicities, and chart reviews increased over time. Over the 8-year period, five additional MOs were hired, two replacing two retiring MOs and three who were completely new. To adjust for the increased number of MOs, we normalized the total volumes based on the total number of cancer treaters (MOs and NPs). We interestingly saw that the increase was primarily due to telephone toxicities and chart reviews, while consults and follow-up visits remained relatively stable with some marginal change depending on the disease site. Marhold et al. have previously published an analysis of patient consult volumes in a large Austrian center, showing a similar rise in consults from 2006 to 2018 [15]. One thought on the lack of proportional growth in consults and follow-ups per Medical Oncologist is that space limitations, including clinic space and chemotherapy chair space, limit the number of patients that can be seen in each clinic. We know treatment in a cancer center is a multifaceted process, requiring personnel (physicians, nursing, pharmacists), space (clinic and chemotherapy chair space), and supportive care (occupational and physiotherapists, dieticians, social workers) provision [16]. Moreover, access to cancer care is directly dependent on the number of providers available [17]. Once space and providers are full, no other patient encounters can occur beyond telephone toxicities and chart checks, as these do not require physical space to be performed. This is also reflected in the encounters seen by an MO, broken down by disease site. No matter which disease site was evaluated, the increase in workload metrics were primarily seen in the chart reviews and telephone toxicities, likely as a result of the evolution of patient assessment during the pandemic. 

With the rapidly evolving treatment landscape and associated complexities, using new consults as the only metric, without consideration of patient encounters in the form of chart reviews, follow-ups, and telephone toxicities (CFT), seems extremely limited. New patient consultations accounted for only 9.8% of all patient encounters in 2021, illustrating the limitation of this one-dimensional metric. Another way of viewing these data is to look at the time spent on patient encounters. For general approximation, consults are a minimum of 1 h in length, follow up visits 30 min, telephone toxicities 10 min, and chart reviews 5 min. Based on the data from 2021, this would equate to 3255 h spent on consults, 6742.5 h spent on follow up visits, 459.8 h on telephone toxicities, and 1151.8 h on chart reviews (a total of 11,609.1). Using those approximations, new consults only accounted for 28% of the total encounter time spent on/with patients in 2021, highlighting the importance of considering other metrics beyond new consults to encompass actual workload. A study by a group in Spain attempted to better-capture medical oncologist workload by assigning a target percentage value for various academic activities (including clinical, research, and educational activities), as well as a proposed time valuation for clinical encounters (for example, time spent for new encounters versus follow-ups) [18]; thus, offering an example of one such potential strategy for workload metric diversification and inclusion.

**Figure 4:**
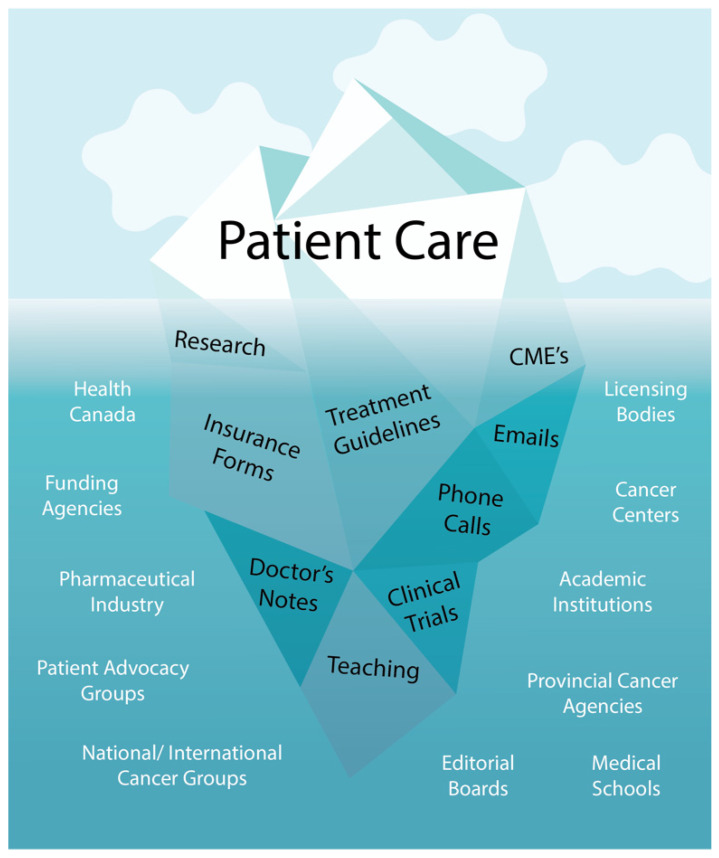
Workload for Oncologists.

Compared to when the CCO was issued, the field of Medical Oncology has undergone revolutionary changes. Many metastatic disease sites with previously limited available treatments now have numerous therapeutic options, with longer survivorship, including melanoma [19], lung, [20], and hepatocellular carcinoma [21] as examples. The advent of both immune checkpoint inhibitors and targeted molecules has expanded the treatment and toxicity landscape far beyond chemotherapy [20]. Additionally, an increasing number of malignancies have more complex treatment regimens relevant to a broader population, for example chemoimmunotherapy in lung cancers regardless of PD-L1 status [20] or multi-agent chemotherapy as standard of care first line treatment in many pancreatic cancers [22]. This was clearly reflected in the patient encounters seen at our individual site over the study period. We have seen both lung and genitourinary disease sites translate into an increase in patient encounters over time, potentially reflecting the availability of new treatments such as immunotherapy, which were not yet regularly integrated into the treatment landscape for colorectal or breast cancers. With newer treatments, physicians may watch their patients more closely for potential toxicities and could employ check-ins with the patient through chart checks and telephone toxicities. Furthermore, with the current national shortage in primary care providers [23], an increase in primary care support required for medical oncology patients will likely continue to contribute to additional encounters. While our data focus specifically on clinical workload, it would be inappropriate not to acknowledge the additional demands on a Medical Oncologist’s time, including time needed for teaching of trainees, research, guideline development, interaction with other institutions, and patient advocacy, to list a few. Patient care is just the tip of the iceberg of a Medical Oncologist’s workload. (Figure 4). 

Despite pandemic restrictions, where patients were relegated to a larger proportion of virtual care, there was still an 7.9% increase in overall patient encounters, with 35% of these occurring in-person. COVID-19 highlighted a trend toward significantly more virtual care provision, a trend that was becoming apparent pre-pandemic. Virtual oncology care was occurring in Nova Scotia before the onset of the COVID-19 pandemic, although its role was limited. Reliance on virtual care increased during the pandemic in the 2020 and 2021 fiscal years, aiming to deliver care whilst maintaining patient and workplace safety. Survey data collected by the Nova Scotia Department of Health and Wellness on the patient experience of virtual care in Nova Scotia during the first months of the COVID-19 pandemic (April to August 2020) found that patients appeared to be highly satisfied with their virtual care experience [24]. Moreover, virtual care has been shown to be highly successful in various oncologic settings and sites, thus supporting an important adaptation suitable to many pre-existing barriers to cancer care access for Canadians, including geographic distance from centers of care, patient morbidity, and cost of travel [24]. With current space and personnel limitations, one of the only avenues for growth is through virtual care. 

The COVID-19 pandemic created new pressures on the system as well, with significant decreases in uptake of screening and surgical delays across the country [9]. In Nova Scotia, breast cancer screening decreased 62% and colorectal cancer screening decreased 65% in 2020 compared to 2019, over the 6-month period from March to December [25]. These interruptions and delays will impact cancer care in the months and years to come, as screening resumes, with more new diagnoses and more advanced diagnoses [26]. 

A workforce planning model has been developed in Radiation Oncology [27], but to our knowledge, nothing similar exists in Medical Oncology. Others are working to create workforce models to help build a stronger future for Canadian Cancer Care, but robust information accounting for all aspects of clinical workload is lacking [6]. This is a critical limitation that needs to be overcome, as development of a robust and meaningful workload assessment and care planning continues. A study from Spain, attempting to better-capture medical oncologist workload, assigned a target percentage value for various academic activities (including clinical, research, and educational activities), as well as a proposed time valuation for clinical encounters (for example, new consults versus follow-ups) [18]. These types of strategies, with percentages or points assigned to clinical and academic tasks, may allow us to capture the full scope of Medical Oncologists’ practice, as well as create a metric that can be adjusted and adapted over time, to meet the ever-evolving demands of cancer care provision. 

We must acknowledge the limitations within our study. As this was a retrospective analysis, we are dependent on the patient encounters logged into OPIS. Any missed or improperly labelled encounters would not have been captured, potentially underrepresenting workload. Moreover, there are workload elements (some mentioned above) that are not directly included in this analysis. Patient phone calls to the nursing/physician team, physician contact with other patient care members, physician pages (the paging system is the primary mode of formal healthcare provider communication at NSHCZ), and others are missing. In February 2020, the Nova Scotia Cancer Centre (and OPIS) began tracking phone calls from patients to their respective cancer care teams (nursing/medical oncologist). For the 2-month period from February through March 2020, 1261 calls were received. This increased from 1 April 2020–31 March 2021, to 8616, and, subsequently, from 1 April 2021 to 31 March 2022, to 13,843. The additional phone calls would increase the total encounters at the cancer center to 47,153. This will be important to capture in the future development of a robust workload metric. As patient encounters are not coded based on disease site, there is no good way of tracking the role of disease sites and treatment options on patient encounters. With the potential adoption of a new electronic patient recording system, we hope to be able to stratify patient encounters to address this more thoroughly in the future. We must finally acknowledge that other centers in Canada may practice differently than Nova Scotia. The size of catchment area, number of visits, the length of visits, and the presence of physician extenders would likely play a role in the outcomes of that province’s analysis. As we are the first to have taken this approach, it would be very interesting to see if similar outcomes are seen in some of the other larger provinces. We would expect a similar trend as space utilization is already currently maximized at many centers, leading to longer wait times, and a reliance on virtual care is becoming more standardized. 

## 5. Conclusions

As the incidence of cancer increases and treatments continue to expand and evolve, Medical Oncologists globally can expect a continued increase in workload. This trend has been identified through the national Canadian Cancer statistics, as well as locally with patient encounter data obtained at our individual site. 

In addition to increasing cancer incidence, the changes instituted at the onset of the COVID-19 pandemic highlight some further proportion of lasting change in the delivery of cancer care, in particular, virtual care delivery. New patient consultation metrics, taken in isolation, do not accurately reflect the current trends in Medical Oncology clinical workload. Creating and implementing a new algorithm that is adaptable to the changing therapeutic landscapes would be a new way to capture workload and could translate into the resource needs required to inform healthcare hiring practices and allocation of space and other resources to optimize patient care. 

Our data are the first to break down MO workload in a Canadian province. The hope is that these data will start the conversation about how to take a critical look at the metrics and resource planning in the country to provide creative solutions to deal with the ever-growing demands on medical oncologists. 

## Figures and Tables

**Table 1:** Total patient encounters by encounter type, 2014–2021.

Year	Consults	Follow-Up	Teletoxicities	Chart Review
**2014**	2228	8902	915	5625
**2015**	2607	9380	623	5900
**2016**	2612	9951	648	6267
**2017**	2779	10,577	794	7244
**2018**	2708	11,096	1356	8971
**2019**	3003	11,203	2182	10,907
**2020**	2824	11,721	2006	12,896
**2021**	3254	13,485	2749	13,822
**% Change**	46.1%	51.5%	200.4%	145.7%

**Table 2:** Changes in encounter visits by MO and by consult compared 2014 to 2020.

	# Visits/MO in 2014	# Visits/MO in 2021	# Encounters/Consult (2014)	# Encounters/ Consult (2021)
**GI**				
Consults	111.5	121.9	----	----
Follow-up	380.7	403.4	3.4	3.3
Teletoxicities	42.9	55.1	0.4	0.5
Chart Review	186.9	343.7	1.7	2.8
**BREAST**				
Consults	101.8	94.9	----	----
Follow-up	391.5	337.1	3.8	3.6
Teletoxicities	49.1	57.7	0.5	0.6
Chart Review	191.9	338.2	1.9	3.6
**GU**				
Consults	123.9	94.5	----	----
Follow-up	614.1	625.6	5.0	6.3
Teletoxicities	52	147.7	0.4	1.5
Chart Review	565	560.7	4.6	5.6
**LUNG**				
Consults	98.2	111.7	----	----
Follow-up	443.4	464.9	4.5	4.2
Teletoxicities	32.4	105.2	0.3	0.9
Chart Review	329	586.2	3.4	5.3

## Data Availability

Not applicable.
